# Rhodotorula silvicola sp. nov., a new yeast species from plant-associated substrates and mushroom

**DOI:** 10.1099/ijsem.0.006836

**Published:** 2025-07-11

**Authors:** Yu-Hua Wei, Liang-Chen Guo, Hai-Yan Zhu, Zhang Wen, Shang-Jie Yao, Xiao-Long You, En-Di Fan, Kyria Boundy-Mills, Irnayuli Sitepu, Feng-Yan Bai, Di-Qiang Wang, Pei-Jie Han

**Affiliations:** 1 State Key Laboratory of Microbial Diversity and Innovative Utilization, Institute of Microbiology, Chinese Academy of Sciences, Beijing 100101, PR China; 2 College of Life Sciences, University of Chinese Academy of Sciences, Beijing 100049, PR China; 3 GuiZhou XiJiu Co., Ltd, Zunyi,Guizhou 564622, PR China; 4 Department of Food Science and Technology, University of California Davis, Davis, CA 95616, USA

**Keywords:** basidiomycetous yeast, phylogeny, taxonomy, *Rhodotorula silvicola* sp. nov.

## Abstract

An orange-coloured yeast strain was recently isolated from rotted leaves in Zunyi City, Guizhou Province, PR China. Phylogenetic analysis revealed that this strain shares identical or similar sequences with no more than two to three nucleotide substitutions in both the 26S rDNA D1/D2 domains and the internal transcribed spacer (ITS) region with three other strains isolated from decayed wood in Indonesia, mushroom in Thailand and a plant of the genus *Opuntia* in the Bahamas. Therefore, these strains are conspecific. And they are most closely related to *Rhodotorula paludigena* but exhibit sequence divergences of 2.1–2.6% in the 26S rDNA D1/D2 domains and 2.9–3.2% in the ITS region. Physiologically, strain CGMCC 2.7770 differs from *R. paludigena* in its ability to assimilate maltose, l-arabinose and citric acid. Both the phylogenetic analysis and phenotypic characteristics indicate that those four strains represent a novel species in the genus *Rhodotorula*, for which the name *Rhodotorula silvicola* sp. nov. is proposed (holotype CGMCC 2.7770^T^).

## Introduction

*Rhodotorula* is a basidiomycete yeast genus within the subphylum *Pucciniomycotina* that was initially established by Harrison in 1928 as an asexual group [1], while the corresponding sexual group *Rhodosporidium* was proposed by Banno in 1967 [2]. However, the genus *Rhodotorula* is polyphyletic based on the molecular phylogenetic analysis and distributed across three classes: *Microbotryomycetes*, *Cystobasidiomycetes* and *Exobasidiomycetes*, which did not accurately reflect the true evolutionary relationships among its species [3,5]. Wang *et al*. performed phylogenetic analyses on 184 pucciniomycetous yeast species including the *Rhodotorula* species which we are concerned with in this study based on the sequences of seven genes, including the small subunit ribosomal DNA (rDNA), the 26S rDNA D1/D2 domains, the internal transcribed spacer (ITS 1 and 2) regions of rDNA including the 5.8S rDNA gene, the nuclear protein-coding genes of the two subunits of DNA polymerase II and the translation elongation factor 1-αand the mitochondrial gene cytochrome b[3,4]. The taxonomic system of these yeasts was consequently revised by Wang *et al*. [3,4]. The revised genus *Rhodotorula* encompasses only 15 species within the *Rhodosporidium* clade, with *Rhodotorula glutinis* designated as the type species [4]. Subsequently, several *Rhodotorula* species were described, including *Rhodotorula aurum*, *Rhodotorula frigidialcoholis*, *Rhodotorula linzhiensis*, *Rhodotorula ngohengohe* and *Rhodotorula sampaioana* [6,10].

Currently, a total of 20 species are recognized in the genus *Rhodotorula*, and among them, six species have been observed to exhibit sexual reproduction, namely, *Rhodotorula babjevae*, *Rhodotorula diobovata*, *Rhodotorula kratochvilovae*, *Rhodotorula paludigena*, *Rhodotorula sphaerocarpa* and *Rhodotorula toruloides* [11,15]. The most notable physiological characteristics of the genus *Rhodotorula* are the lack of the inability to synthesize starch-like compounds and the positive reactions for the diazonium blue B test and urease production [1]. Notably, species in the genus *Rhodotorula* are capable of synthesizing various carotenoids, including *β*-carotene and red pigments, which have found extensive application in the food, cosmetic and pharmaceutical industries [16].

During a survey of yeast diversity in Xijiu town, a historical Baijiu production region in Guizhou Province, PR China, a strain with orange colonies was isolated from rotted leaves. Based on conventional physiological tests and sequence comparisons of the D1/D2 domains and the ITS region, the strain was identified as a novel *Rhodotorula* species, namely, *Rhodotorula silvicola* sp. nov. Our molecular phylogenetic analysis showed that three strains isolated from substrates in the Bahamas, Thailand and Indonesia have identical or similar D1/D2 and ITS sequences with *R. silvicola* sp. nov., which indicated that they are conspecific.

## Methods

### Sampling and yeast isolation

Rotten leaf samples were collected from the environment surrounding Xijiu Distillery in Zunyi City, Guizhou Province, PR China, in September 2023. Five grams of leaves were placed in sterile sampling bags, transported to the laboratory and suspended in a 100 ml sterile conical flask containing 30 ml of sterile water. The conical flask containing the suspensions was placed in a 25 °C shaking incubator at 200 g for 30 min to ensure thorough and uniform dispersion of yeast cells from the sample in the sterile water, then diluted to 10^−3^, 10^−4^ and 10^−5^. An aliquot of 200 µl from each dilution was plated on yeast peptone dextrose agar (YPD: 1% yeast extract, 2% peptone, 2% dextrose and 2% agar) supplemented with 0.02% chloramphenicol and 0.2% acetic acid. The plates were incubated at 17 °C for 3–5 days and evaluated daily for colony formation. The selected orange colony yeast strain CGMCC 2.7770 was streaked onto separate YPD agar plates for purification. Following purification, the strain was suspended in YPD broth containing 20% (v/v) glycerol and stored at −80 °C for future use.

### Phenotypic characterization

Morphological, physiological and biochemical characteristics were analysed following established protocols [17]. Carbon and nitrogen compound assimilation tests were conducted using liquid media. To study the formation of sexual structures, strain CGMCC 2.7770 was inoculated on corn meal agar (CMA: 2.5% corn starch and 2% agar), potato dextrose agar (PDA: 20% potato infusion, 2% glucose and 2% agar), yeast extract-malt extract agar (YM: 1% glucose, 0.3% yeast extract, 0.3% malt extract, 0.5% peptone and 2% agar), yeast carbon base supplemented with 0.01% ammonium sulphate (YCBAS) agar and V8 agar (10% V8 juice and 2% agar). The plates were incubated at 20 °C for up to 2 months with periodic observations for the sexual structures. The formation of hyphae was studied by inoculating the activated strain on CMA and PDA, which were incubated at 20 °C for 10 days with a cover glass to create an oxygen-free environment.

### Molecular phylogenetic analysis

A sesame seed-sized quantity of fresh yeast cells was transferred to 70 µl of sterile 0.1 M sodium hydroxide solution, where they were subjected to lysis at 98 °C for 15 min to extract yeast genomic DNA. The fragment encompassing the nuclear ribosomal ITS region and the 26S rDNA D1/D2 domains was amplified and sequenced following established protocols, with the D1/D2 domains amplified using primers NL1 and NL4 and the ITS region amplified using primers ITS1 and ITS4 [18]. Specifically, amplifications were performed in a 25 µl reaction volume consisting of 22 µl of 1.1×T3 Super PCR Mix (Tsingke Biotech Co., Beijing, China), 1 µl of DNA template and 1 µl of each primer. The PCR programme was as follows: initial denaturation at 98 °C for 2 min; 35 cycles of 98 °C for 10 s, 55 °C for 15 s and 72 °C for 15 s; and a final extension of 72 °C for 5 min. The PCR products were purified using the PCR product purification kit (Sangon Biotech, Shanghai, China). Both DNA strands were sequenced using the ABI BigDye Terminator Cycle Sequencing kit (Applied Biosystems, CA, USA) and run on an ABI 3730 automated DNA sequencer (Applied Biosystems, CA, USA) according to the manufacturer’s instructions.

Sequence alignment was carried out using MAFFT v.7 [19], with manual adjustments made as necessary in mega v.7 [20]. Phylogenetic analyses were performed using evolutionary distances calculated with Kimura’s two-parameter model [21], applying the neighbour-joining method in mega v.7 [20,22]. For the concatenated D1/D2 and ITS sequences, the maximum likelihood analyses were performed using RAxML v.8 [23]. Bootstrap support was assessed through 1,000 replicates [24].

## Results and discussion

### Phylogenetic analysis

**Fig. 1.**
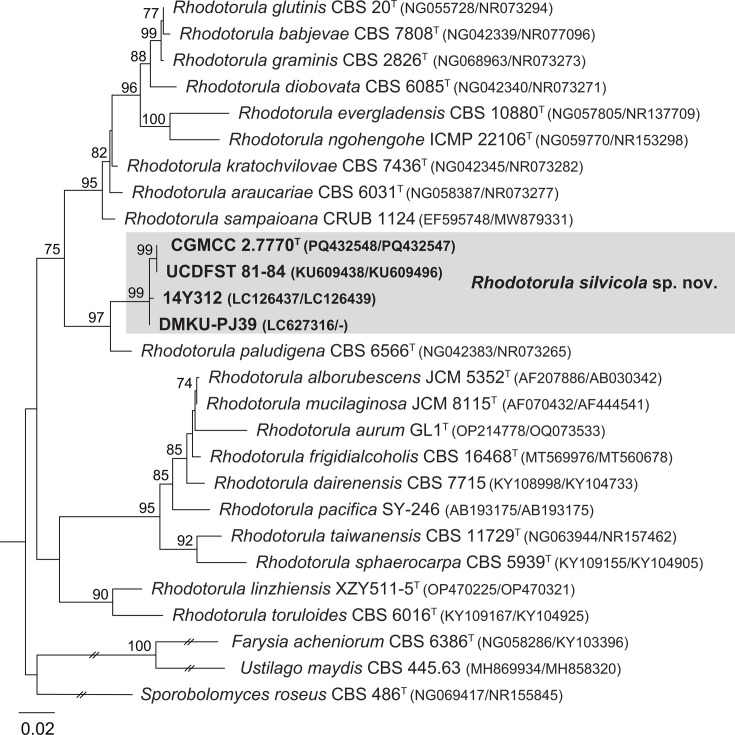
Maximum likelihood phylogenetic tree based on the combined sequences of the 26S rDNA D1/D2 domains and the ITS region showing the phylogenetic placement of the novel species *R. silvicola* sp. nov. Bootstrap values≥70% are shown on the branches of the tree. Three species, *Farysia acheniorum*, *Ustilago maydis* and *Sporobolomyces roseus*, are used as the outgroup. Type strains are denoted with a superscripted ‘T’. Bar, 0.02 substitutions per nucleotide position.

Strain CGMCC 2.7770^T^ was primarily identified through a blast search in GenBank using the D1/D2 and ITS sequences as queries. The D1/D2 sequence blast showed that strain CGMCC 2.7770^T^ possesses identical or similar D1/D2 sequences (with no more than three base mismatches) to three strains from other sources: strain UCDFST 81-84 isolated from a plant of the genus *Opuntia* in the Bahamas, strain 14Y312 isolated from decayed wood in Indonesia and strain DMKU-PJ39 isolated from mushrooms in Thailand. These strains were previously identified as *Rhodotorula aff. paludigenum*, *Rhodosporidium* sp. and *Rhodotorula* sp., respectively. Among them, strain UCDFST 81-84 shares an identical ITS sequence with CGMCC 2.7770, while strain 14Y312 differs from CGMCC 2.7770 by two base substitutions in the ITS region, and strain DMKU-PJ39 lacks the ITS sequence. The phylogenetic tree based on the 26S rDNA D1/D2 domains or ITS region sequences showed that strain CGMCC 2.7770 and those three strains from different countries form a clade closely related to *R. paludigena* (Figs S1 and S2, available in the online Supplementary Material). The above analyses indicate that strains CGMCC 2.7770, UCDFST 81-84, 14Y312 and DMKU-PJ39 are conspecific. The phylogenetic analyses based on the 26S rDNA D1/D2 domains and the ITS region sequences confirmed the affinity of the CGMCC 2.7770^T^ group to *R. paludigena* (Fig. 1). In addition, two studies clearly indicated that UCDFST 81-84 is a potential new species and is closely related to *R. paludigena* [25,26], which is consistent with this study. Meanwhile, strain CGMCC 2.7770 differs from the type strain of *R. paludigena* by 14 (2.6%, 12 substitutions and 2 gaps) and 15 (2.9%, 12 substitutions and 3 gaps) mismatches in the 26S rDNA D1/D2 domains and ITS region, respectively. The above results indicate that the CGMCC 2.7770 group represents a novel species of the genus *Rhodotorula*, for which the name *R. silvicola* sp. nov. is proposed.

### Phenotypical characteristics and ecology

The morphological, biochemical and physiological characteristics of strain CGMCC 2.7770 isolated from decaying leaves in China and strain UCDFST 81-84 collected from a plant of the genus *Opuntia* in the Bahamas were determined. Physiologically, the new species *R. silvicola* is distinguished from its closely related species *R. paludigena* by its capacity to grow on glucose-yeast extract agar with concentrations as high as 50% (w/v) and 60% (w/v), and by its inability to assimilate maltose, l-arabinose, d-ribose and citric acid (Table 1). Additionally, *R. silvicola* can be distinguished from *R. kratochvilovae*, *Rhodotorula araucariae* and * R. sampaioana* by its inability to assimilate citric acid (Table 1).

**Table 1.** Salient phenotypical characteristics distinguishing *R. silvicola* sp. nov. from the closely related species

Species	Assimilation				Growth	Reference
	Maltose	l-Arabinose	d-Ribose	Citric acid	50% glucose-yeast extract agar	
*R. silvicola* sp. nov.	–	–	–	–	+	This study
*R. paludigena*	+/L	+/L	+/L	+/W	–	[11]
*R. kratochvilovae*	+	–	–	+/W	Not available	[14]
*R. araucariae*	–	–	–	+/L	Not available	[28]
*R. sampaioana*	–	–	–	+	+/W	[8]

–, negative; +, positive; L, laten; W, weak.

The species of the genus *Rhodotorula* demonstrate wide ecological adaptability and have been isolated from various natural substrates and environments. These include plant-associated substrates, i.e. leaves [12,27], decayed bark [28] and wood [10] In addition, they have also been found in marine and freshwater habitats [8,13, 15, 29,31], gold mine soil [9], marshes [11], permafrost [7] and feathers [6]. Three strains of *R. silvicola* sp. nov. were isolated from plant-associated substrates, i.e. rotted leaves or wood, or a plant of the genus *Opuntia*, but one strain from mushroom, which is the first reported.

## Description of *Rhodotorula silvicola* Y.H. Wei, L.C. Guo, P.J. Han, D.Q. Wang and F.Y. Bai sp. nov.

*Rhodotorula silvicola* [sil.vi'co.la. L. fem. n. *silva*, forest; L. suff. -*cola* (from L. masc./fem. n. *incola*, inhabitant, dweller; N.L. fem. n. *silvicola*, forest dweller)].

**Fig. 2.**
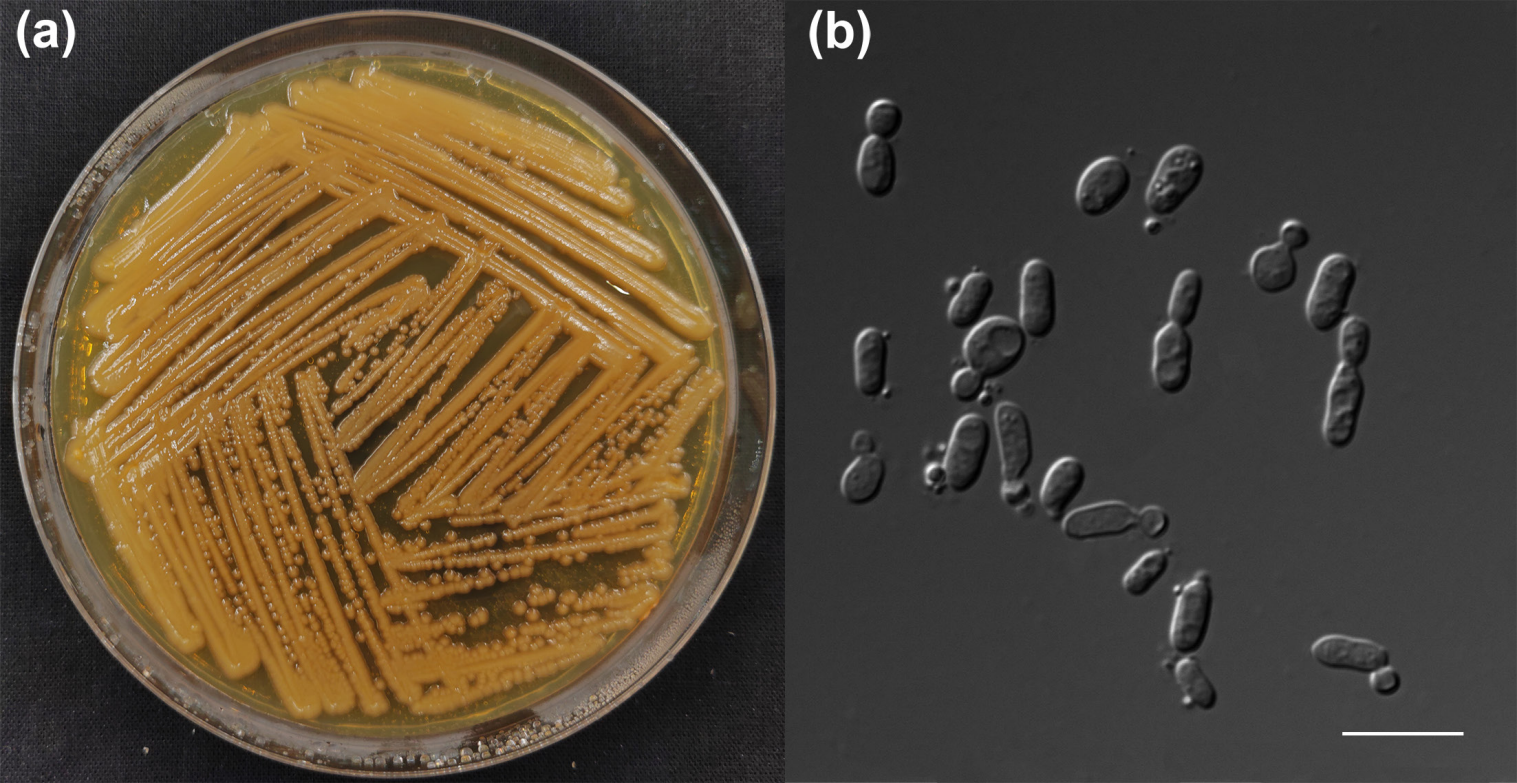
The morphology of *R. silvicola* sp. nov. strain CGMCC 2.7770^T^ observed on YPD agar after 1 week at 20 °C includes colony morphology (**a**) and vegetative cells (**b**). Bars: 10 µm.

Culture characteristics: after growth on YPD agar for one week at 20 °C, cultures are orange, butyrous, smooth and glossy (Fig. 2a), and the vegetative cells are ovoid, ellipsoidal or elongate (1.3–6.7×1.1–4.4 µm) and occur singly or as budded pairs; reproduction is primarily by unilateral budding (Fig. 2b). After 1 month of incubation in YM broth at 20 °C, an orange sediment and pellicle are formed. Neither pseudohyphae nor true hyphae are formed on CMA and PDA after 10 days at 20 °C. Additionally, sexual structure was not observed in the single and pairwise mixed strain cultures of strain CGMCC 2.7770 and strain UCDFST 81-84 on different media, including CMA, PDA, YM, YCBAS and V8, after incubation at 20 °C for 2 months.

Physiological and biochemical characteristics: glucose is not fermented. Glucose, sucrose, cellobiose, trehalose, raffinose (weak), xylose (weak), ethanol, glycerol (slow), ribitol, galactitol, mannitol, glucitol, salicin, succinate acid (weak) and xylitol (weak) are assimilated as sole carbon sources. Galactose, l-sorbose, maltose, lactose, melibiose, melezitose, inulin, soluble starch, l-arabinose, d-arabinose, d-ribose, rhamnose, d-glucosamine, methanol, erythritol, methyl-*α*-glucoside, glucuronic acid, dl-lactate, citric acid, inositol, hexadecane and *N*-acetyl-d-glucosamine are not assimilated as sole carbon sources. Ammonium sulphate, potassium nitrate, sodium nitrite and ethylamine are assimilated as sole nitrogen sources. Cadaverine and l-lysine are not assimilated as sole nitrogen sources. Growth in vitamin-free medium is positive. The diazonium blue B reaction is positive. Extracellular starch-like compounds are not produced. Urease activity is positive. Growth on 10% (w/v) sodium chloride plus 5% (w/v) glucose medium is negative. Growth on 50% (w/v) glucose-yeast extract agar and 60% (w/v) glucose-yeast extract agar is positive. Growth on YPD agar is positive at 30 °C but negative at 37 °C.

The holotype, CGMCC 2.7770^T^ (original strain number 1017-17-3), was isolated from rotten leaves from Zunyi City, Guizhou Province, PR China, in September 2023, and has been deposited in a metabolically inactive state in the China General Microbiological Culture Collection Center, Beijing, PR China. The ex-type culture has been deposited in the Japan Collection of Microorganisms (JCM), Koyadai, Japan, as JCM 36980. The GenBank accession number for the 26S rDNA D1/D2 domains and the ITS region sequences of the type strain 1017-17-3 is PQ432548 and PQ432547, respectively. The Mycobank number is MB 859571.

## Supplementary material

10.1099/ijsem.0.006836 Uncited Supplementary Material 1.

## Abbreviations

CMA: corn meal agar
ITS: internal transcribed spacer
JCM: Japan Collection of Microorganisms
PDA: potato dextrose agar
rDNA: ribosomal DNA
YCBAS: yeast carbon base supplemented with 0.01% ammonium sulphate agar
YM: yeast extract-malt extract
YPD: yeast peptone dextrose

## References

[1] Sampaio JP, Kurtzman CP, Fell JW, Boekhout T (1928). The Yeasts, A Taxonomic Study.

[2] Sampaio JP, Kurtzman CP, Fell JW, Boekhout T (1967). The Yeasts, A Taxonomic Study.

[3] Wang QM, Groenewald M, Takashima M, Theelen B, Han PJ (2015). Phylogeny of yeasts and related filamentous fungi within *Pucciniomycotina* determined from multigene sequence analyses. Stud Mycol.

[4] Wang QM, Yurkov AM, Göker M, Lumbsch HT, Leavitt SD (2015). Phylogenetic classification of yeasts and related taxa within *Pucciniomycotina*. Stud Mycol.

[5] McNeill J, Barrie FR, Buck WR, Demoulin V, Greuter W (2012). International Code of Nomenclature for Algae, Fungi, and Plants (Melbourne Code).

[6] Crous PW, Wingfield MJ, Burgess TI, Hardy GESJ, Barber PA (2017). Fungal planet description sheets: 558-624. Persoonia.

[7] Touchette D, Altshuler I, Gostinčar C, Zalar P, Raymond-Bouchard I (2022). Novel Antarctic yeast adapts to cold by switching energy metabolism and increasing small RNA synthesis. ISME J.

[8] Tiwari S, Baghela A, Libkind D (2021). *Rhodotorula sampaioana* f.a., sp. nov., a novel red yeast of the order *Sporidiobolales* isolated from Argentina and India. Antonie van Leeuwenhoek.

[9] Thi Nguyen KC, Truong PH, Ho CT, Le CT, Tran KD (2023). Copper tolerance of novel *Rhodotorula* sp. yeast isolated from gold mining ore in Gia Lai, Vietnam. Mycobiology.

[10] Jiang YL, Bao WJ, Liu F, Wang GS, Yurkov AM (2024). Proposal of one new family, seven new genera and seventy new basidiomycetous yeast species mostly isolated from Tibet and Yunnan provinces, China. Stud Mycol.

[11] Fell JW, Statzell Tallman A (1980). *Rhodosporidium paludigenum* sp. nov., a basidiomycetous yeast from intertidal waters of South Florida. Int J Syst Bacteriol.

[12] Golubev W (1993). *Rhodosporidium babjevae*, a new heterothallic yeast species (*Ustilaginales*). Syst Appl Microbiol.

[13] Newell SY, Hunter IL (1970). *Rhodosporidium diobovatum* sp. n., the perfect form of an asporogenous yeast (*Rhodotorula* sp.). J Bacteriol.

[14] Hamamoto M, Sugiyama J, Komagata K (1988). *Rhodosporidium kratochvilovae* sp. nov. a new basidiomycetous yeast species. J Gen Appl Microbiol.

[15] Newell SY, Fell JW (1970). The perfect form of a marine-occurring yeast of the genus *Rhodotorula*. Mycologia.

[16] Mortensen A (2006). Carotenoids and other pigments as natural colorants. Pure Applied Chem.

[17] Kurtzman CP, Fell JW, Boekhout T, Kurtzman CP, Fell JW, Boekhout T (2011). The Yeasts, A Taxonomic Study.

[18] Bai FY, Zhao JH, Takashima M, Jia JH, Boekhout T (2002). Reclassification of the *Sporobolomyces roseus* and *Sporidiobolus pararoseus* complexes, with the description of *Sporobolomyces phaffii* sp. nov. Int J Syst Evol Microbiol.

[19] Katoh K, Standley DM (2013). MAFFT multiple sequence alignment software version 7: improvements in performance and usability. Mol Biol Evol.

[20] Kumar S, Stecher G, Tamura K (2016). MEGA7: molecular evolutionary genetics analysis version 7.0 for bigger datasets. Mol Biol Evol.

[21] Kimura M (1980). A simple method for estimating evolutionary rates of base substitutions through comparative studies of nucleotide sequences. J Mol Evol.

[22] Lachance MA (2022). Phylogenies in yeast species descriptions: in defense of neighbor-joining. Yeast.

[23] Stamatakis A (2014). RAxML version 8: a tool for phylogenetic analysis and post-analysis of large phylogenies. Bioinformatics.

[24] Felsenstein J (1985). Confidence limits on phylogenies: an approach using the bootstrap. Evolution.

[25] Garay LA, Sitepu IR, Cajka T, Cathcart E, Fiehn O (2017). Simultaneous production of intracellular triacylglycerols and extracellular polyol esters of fatty acids by *Rhodotorula babjevae* and *Rhodotorula aff. paludigena*. J Ind Microbiol Biotechnol.

[26] Garay LA, Sitepu IR, Cajka T, Chandra I, Shi S (2016). Eighteen new oleaginous yeast species. J Ind Microbiol Biotechnol.

[27] Huang CH, Lee FL, Tien CJ, Hsieh PW (2011). *Rhodotorula taiwanensis* sp. nov., a novel yeast species from a plant in Taiwan. Antonie van Leeuwenhoek.

[28] Grinbergs J, Yarrow D (1970). *Rhodotorula araucariae* sp. n. Antonie van Leeuwenhoek.

[29] Fell JW, Statzell-Tallman A, Scorzetti G, Gutiérrez MH (2011). Five new species of yeasts from fresh water and marine habitats in the Florida Everglades. Antonie van Leeuwenhoek.

[30] Gadanho M, Sampaio JP, Spencer-Martins I (2001). Polyphasic taxonomy of the basidiomycetous yeast genus *Rhodosporidium*: *R. azoricum* sp. nov. Can J Microbiol.

[31] Nagahama T, Hamamoto M, Horikoshi K (2006). *Rhodotorula pacifica* sp. nov., a novel yeast species from sediment collected on the deep-sea floor of the north-west Pacific Ocean. Int J Syst Evol Microbiol.

