# Supplementary material for: Rhodotorula silvicola sp. nov., a new yeast species from plant-associated substrates and mushroom
Source: Int J Syst Evol Microbiol. 2025 Jul 11;75(7):006836. doi: 10.1099/ijsem.0.006836 (PMC12247233; doi:10.1099/ijsem.0.006836)
Supplement: Uncited Supplementary Material 1. [file ijsem-75-06836-s001.pdf]

Supplementary materials

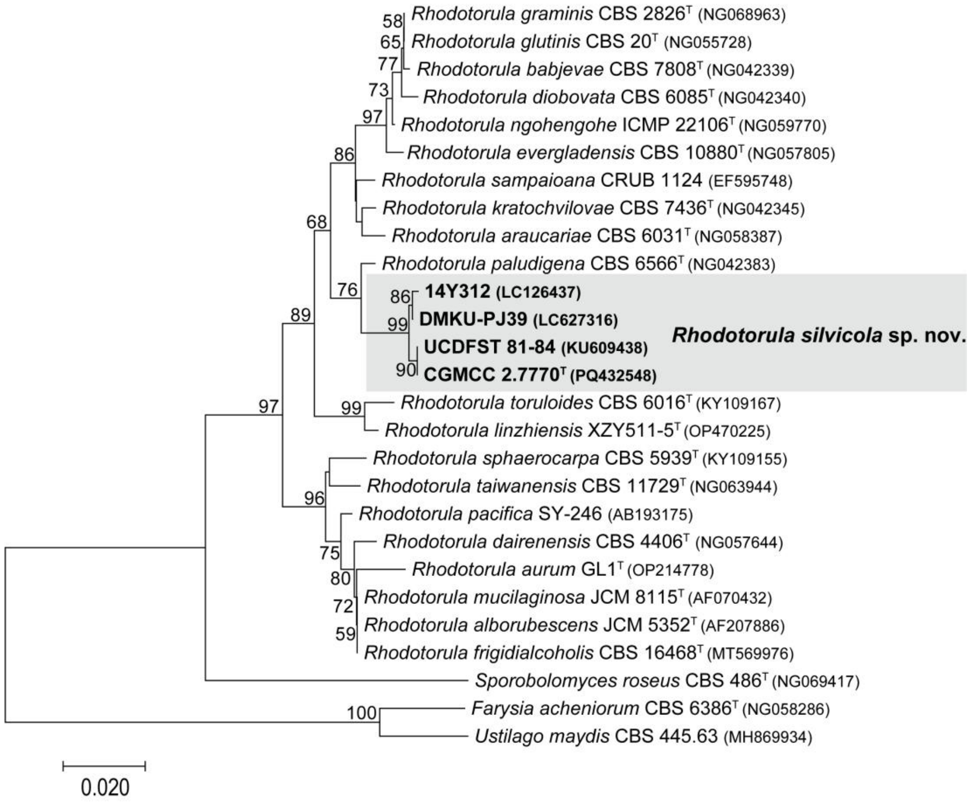

Fig. S1 Neighbor-Joining phylogenetic tree based on the 26S rDNA D1/D2 domains showing the phylogenetic placement of the novel species *Rhodotorula silvicola* sp. nov. Bootstrap values ≥ 50% are shown on the branches of the tree. Species *Sporobolomyces roseus*, *Farysia acheniorum* and *Ustilago maydis* are used as the outgroup. Type strains are denoted with a superscripted 'T'. Bar, 0.02 substitutions per nucleotide position.

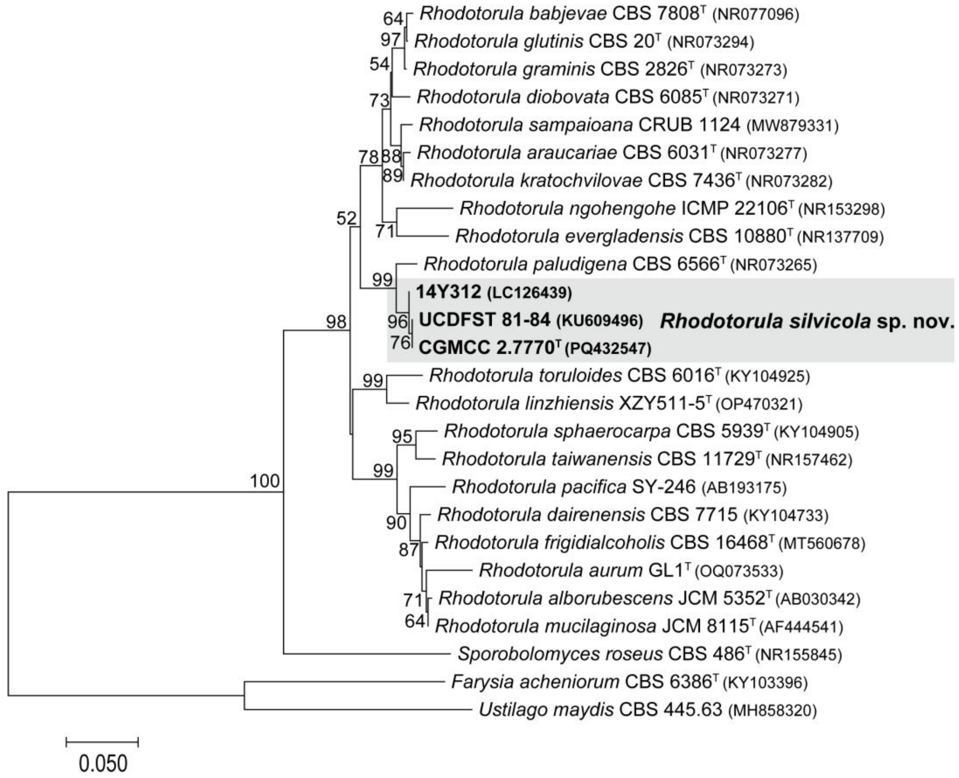

Fig. S2 Neighbor-Joining phylogenetic tree based on the ITS region showing the phylogenetic placement of the novel species *Rhodotorula silvicola* sp. nov. Bootstrap values ≥ 50% are shown on the branches of the tree. Species *Sporobolomyces roseus*, *Farysia acheniorum* and *Ustilago maydis* are used as the outgroup. Type strains are denoted with a superscripted ‘T’. Bar, 0.05 substitutions per nucleotide position.
